# Connaissances et comportements de 50 familles congolaises concernées par la drépanocytose: une enquête locale

**DOI:** 10.11604/pamj.2018.29.24.12276

**Published:** 2018-01-11

**Authors:** Benoît Mbiya Mukinayi, Didier Kalombo Kalenda, Stéphanie Mbelu, Béatrice Gulbis

**Affiliations:** 1 Faculté de Médecine, Université de Mbujimayi, Mbujimayi, République Démocratique du Congo; 2 Hôpital Erasme, Université Libre de Bruxelles, Bruxelles, Belgique

**Keywords:** Drépanocytose, Mbujimayi, RDC, connaissances, attitudes socio-économique, Sickle cell adisease, Mbujimayi, Democratic Republic of the Congo, awareness, socioeconomic attitudes

## Abstract

**Introduction:**

La drépanocytose est très fréquente en République Démocratique du Congo (RDC). Cette étude évalue le niveau de connaissance et les comportements de familles concernées ainsi que l’impact de cette maladie dans leur quotidien.

**Méthodes:**

C’est une étude transversale, sur un échantillon non aléatoire, menée du 15 juin au 15 août 2015 auprès de 50 familles concernées par la drépanocytose à Mbujimayi en RDC.

**Résultats:**

Sur 50 familles étudiées, 22 familles avaient plus d’un enfant drépanocytaire. L’âge médian au diagnostic était de 1 an. Le diagnostic était clinique dans 42% (21) des cas. Chaque premier enfant drépanocytaire faisait en moyenne annuellement 3 crises douloureuses, 4 épisodes de fièvre, recevait 2 transfusions sanguines et était hospitalisé 3 fois. 62% (31) des familles n’avaient pas un revenu mensuel suffisant pour la prise en charge de leur(s) enfant(s), 96% (48) des familles souhaite la création d’un centre de référence de prise en charge et 94% (47) acceptent de s’y abonner si le montant annuel est inférieur à 100$.

**Conclusion:**

Le niveau des connaissances dans les familles concernées par la drépanocytose à Mbujimayi est faible. La création d’un centre de référence et la possibilité d’un montant fixe annuel pour la prise en charge des patients est une stratégie qui peut être mise en œuvre à Mbujimayi afin d’améliorer la prise en charge des enfants la drépanocytaires.

## Abstract (en)

**Introduction:**

Sickle cell disease is a very common disease in the Democratic Republic of the Congo, but it is poorly known despite having an impact on the morbi-mortality. Our study aimed to evaluate the extent of awareness and attitudes of families affected by sickle cell disease as well as the impact of this disease in their daily lives.

**Methods:**

We conducted a cross-sectional study of a unique non-random sampling from 50 families affected by sickle cell disease in Mbujimayi, Democratic Republic of the Congo, from 15 June to 15 August 2015. Participants were interviewed using a standardized questionnaire.

**Results:**

This study focuses on 50 families affected by sickle cell disease; medical characteristics were found only in first children affected by sickle cell disease in each household. Less than 10% of families had minimally ill children. Fifty families were interviewed, of whom 22 had more than one child with sickle cell disease. The average age at diagnosis was 1 years. Diagnosis was based on clinical examination in 42% (21) of cases. Each first child affected by sickle cell disease had an average of 3.4 crises per year, 4 episodes of fever per year, received an average of 1.9 transfusions per year and was hospitalized an average of 3 times per year. Thirty-one families (62%) didn’t have sufficient monthly income to help their children to manage sickle cell disese, 48 (96%) families hoped that a reference sickle cell centre would be established in Mbujimayi and 47 (94%) would accept to subscribe to health insurance if the annual amount were between $50 and $100.

**Conclusion:**

The extent of awareness among families directly affected by sickle cell disease in Mbujimayi, Democratic Republic of the Congo, is low. This has a direct impact on the management of children with sickle cell disease. The socioeconomic status of these families is also a factor which should be taken into account. The establishement of a reference center and the possibility to fix an annual amount for the treatment of patients would be a strategic approach to implement the awareness and the attitudes of families in relation to this disease having a direct impact on the morbi-mortality of patients.

## Introduction

La drépanocytose est une des maladies héréditaires récessives les plus fréquentes à travers le monde et en particulier en Afrique sub-saharienne. Elle affecte plus de 6 millions de nouveau-nés par an qui ont des traits drépanocytaires [1-3]. En RDC, les données épidémiologiques récentes ont montré que 2% des nouveau-nés sont homozygotes pour l’hémoglobine S et environ 40.000 naissances d'enfants drépanocytaires sont estimées chaque année, tandis que dans la population adulte le portage du trait s'élève à environ 25% [4, 5]. Si ce chiffre est significatif au point de vue épidémiologique, la maladie reste peu connue avec pour conséquence une forte mortalité dans un pays à ressources limitées [6, 7].

La couverture du programme national de lutte contre la drépanocytose (PNLCD) en RDC reste néanmoins largement insuffisante. Le système de soins de santé en RDC est organisé en 3 niveaux selon un modèle pyramidal: niveau central, niveau intermédiaire et niveau périphérique. Le niveau central est le niveau normatif et régulateur; le niveau intermédiaire a un rôle d’appui et d’accompagnement ; le niveau périphérique est la Zone de Santé (ZS) qui constitue le niveau opérationnel des activités de soins de santé primaires. Le PNLCD a intégré la prise en charge dans 20 ZS de 4 provinces (Bandundu, Bas Congo, Katanga et Kinshasa) sur le 515 que compte le pays. A l’instar d’autres programmes nationaux de lutte contre les maladies d’utilités de santé publique comme le VIH/SIDA, la tuberculose, le paludisme avec des activités de sensibilisation de la communauté, de renforcement des préventions primaires couplées à des actions médicales, le PNLCD n’est pas encore opérationnel à Mbujimayi au Kasaï-Oriental [8]. Le PNLCD a pour mandat la prévention de la transmission de la drépanocytose; l’amélioration de l’état de connaissance des populations sur la drépanocytose; la réduction de l’impact de la drépanocytose sur l’individu, la famille et la société; et l’organisation et la régulation de la prise en charge des drépanocytaires [8]. Il est donc primordial que les familles ayant au moins un enfant drépanocytaire puissent être accompagnées efficacement par le personnel médical et les communautés, car ils sont plus susceptibles d'adopter des comportements protecteurs pour eux-mêmes et pour leurs enfants malades, lorsqu'ils disposent d'une information appropriée sur la drépanocytose. Aucune étude n’a été réalisée localement au Kasaï. On ignore l’importance réelle de la drépanocytose et son impact dans les familles impliquées en particulier à Mbujimayi. L'objectif de notre étude est de réaliser une évaluation du niveau de connaissance et des comportements vis-à-vis de la drépanocytose de familles directement concernées et ainsi que de l’impact de cette maladie dans leur quotidien.

## Méthodes

Cette étude a été réalisée dans les cinq communes de la ville de Mbujimayi (Muya, Dibindi, Kanshi, Diulu et Bipemba), Chef-lieu de la Province du Kasaï oriental au centre-Est de la RDC. L’étude transversale a été réalisée sur une période de 2 mois (15 juin au 15 août 2016). Le recrutement a été obtenu sur base de l’identification des familles grâce aux relais communautaires et sur base de la déclaration libre du tuteur répondant. Nous nous sommes arrêtés à 50 familles suite à une redondance des réponses. Les interviews ont été menées par 15 étudiants en médecine ayant bénéficié d’une formation sur la conduite de l’étude. Chaque famille interviewée était représentée par un tuteur répondant qui était soit le père (22%), soit la mère (64%) ou tout autre adulte > 18 ans faisant partie de la famille (14%).

### Population étudiée

Etait inclus, tout tuteur de ≥ 18 ans d’un ou de plusieurs patients drépanocytaires âgés de plus de 6 mois et ayant consenti à participer à l’étude. A été exclu, tout tuteur répondant d’un enfant drépanocytaire ≥ 6 mois absent du domicile lors du passage des enquêteurs. La taille de la population est basée sur le nombre de tuteurs répondants. Cette étude a été approuvée par l’Université de Mbujimayi et a fait l’objet d’une lettre de recommandation. Un consentement libre et éclairé a été obtenu de la part des enquêtés. Les données récoltées étaient analysées d’une manière anonyme.

### Questionnaire

Le questionnaire a été établi par une équipe de la faculté de médecine de l’université de Mbujimayi et validé par une étude pilote sur 15 patients. Les questions ont abordé les points suivants: les données personnelles des malades et des familles (nombre de malades par famille, âge du 1^er^ enfant drépanocytaire par famille, âge du diagnostic du 1^er^ enfant drépanocytaire, etc.); le degré de sévérité de la maladie au sein de chaque famille; les connaissances du tuteur répondant (père ou mère) vis-à-vis de la maladie; les attitudes de prise en charge; les répercussions de la maladie sur le plan social.

Le nombre estimé des crises vaso-occlusives par an, a été défini comme le nombre d’épisodes douloureux ayant nécessité une prise en charge dans une structure de santé. L’évaluation des connaissances, attitudes et pratiques sur la drépanocytose a été faite sur base des critères d’évaluation des connaissances des enquêtés sur la drépanocytose classées en bonne connaissance, assez bonne connaissance, passable et nulle. Seules des questions fermées ont été posées, soit dichotomiques (ex. sexe), ou nominale (ex. niveau d’éducation) ou à choix multiples (ex. niveau de connaissances).

### Analyse statistique

L’analyse statistique des données collectées a été faite à l’aide du logiciel EPI Info (USA, CDC Atlanta, 2008) version 5.0. Les données ont, selon le cas, été représentées par: la moyenne et leur intervalle de confiance à 95% pour les paramètres numériques; les fréquences absolues ou relatives pour les paramètres caractériels.

Le test paramétrique d’Anova a été utilisé pour comparer les moyennes avec un intervalle de confiance à 95%. Les valeurs p < 0,05 étaient considérées comme statistiquement significatives.

## Résultats

Un total de 50 interviews auprès d’un répondant parmi les familles a été collecté. Le nombre total d’enfants était de 271 parmi lesquels 82 (30%) enfants étaient drépanocytaires. Pour les enfants drépanocytaires, 60% (49) sont scolarisés avec une répartition égale entre filles et garçons et 14% (11) ont abandonné leur scolarisation.

### Drépanocytose: connaissances, attitudes et pratiques

Les connaissances, attitudes et pratiques des 50 familles inclues dans l’étude sont rapportées dans les Tableau 1 et Tableau 2.

**Tableau 1:** Evaluation du niveau des connaissances des ménages impliqués par la drépanocytose (n= 50)

	**N**	**%**
**Niveau de connaissance sur la cause de la drépanocytose**		
Bonne connaissance	22	44
Assez bonne connaissance	4	8
Passable	8	16
Connaissance nulle	16	32
**Niveau de connaissance sur le mode de transmission de la drépanocytose**		
Héréditaire	22	44
Un des parents	3	6
Mauvais sort	9	18
Ne sait pas ou autres réponses non exactes	16	32
**Niveau de connaissance sur les complications de la drépanocytose**		
Bonne connaissance	3	6
Assez bonne connaissance	18	36
Passable	18	36
Connaissance nulle	11	22
**Niveau de connaissance sur la durée de la maladie**		
Bonne connaissance	22	44
Connaissance nulle	28	56
**Niveau de connaissance sur les facteurs favorisants les crises**		
Bonne connaissance	4	8
Assez bonne connaissance	11	22
Passable	14	28
Connaissance nulle	21	42

**Tableau 2:** Evaluation des attitudes et pratiques des ménages impliqués par la drépanocytose (n= 50)

Information sur le diagnostic de la drépanocytose de leur enfant	
Oui	74%
Non	26%
**Connaissance des médicaments utilisés à domicile en cas de fièvre du patient drépanocytaire**	
Bonne connaissance	44%
Assez bonne connaissance	14%
Connaissance nulle	42%
**Conduite à tenir adoptée quand le patient drépanocytaire fait de la fièvre à domicile**	
Assez bien	23,7%
Nulle	76,3%
**Association d’autres traitements utilisés pour traiter la drépanocytose**	
Traitement traditionnel	52%
Prière	8%
Pas d’association à d’autres traitements	40%
**Motivation d’amener le patient drépanocytaire à la consultation de suivi médical de contrôle**	
Oui, seulement si épisode aigu	80%
Oui, selon le rendez-vous du médecin même en absence des crises	12,4%
Non	7,6%
**Observation de la prophylaxie contre l’anémie et les infections chez les patients drépanocytaires**	
Acide folique et pénicilline orale régulièrement	0%
Acide folique et amoxicilline régulièrement	2%
Acide folique seul, mais irrégulièrement	26%
Pénicilline orale ou amoxicilline irrégulièrement	2%
Aucune prophylaxie ou produit indigène, antidouleur seulement	70%
**Notion d’antécédent de la prophylaxie à l’hydroxyurée contre les crises drépanocytaires**	
Oui	10%
Non	90%

Les patients ayant une histoire de prophylaxie à l’hydroxyurée auraient au moins pris de l’hydroxyurée une fois au court de leur prise en charge, mais actuellement aucun patient n’est sous prophylaxie à l’hydroxyurée.

### Situation médicale des premiers enfants drépanocytaires

L’âge moyen du diagnostic de la drépanocytose est de 2 ans (médiane = 1 an), qui va de la naissance et à un maximum de 15 ans. La majorité d’enfants drépanocytaires ont été diagnostiqué avant 59 mois de vie et le type de diagnostic était majoritairement clinique (42%). L’électrophorèse de l’hémoglobine a été réalisée dans 38% des cas et le test de falciformation dans 20% des cas (Tableau 3). Le Tableau 4 montre que chaque premier enfant drépanocytaire faisait en moyenne 3 crises vaso-occlusives par an, environ 4 épisodes des fièvres par an, recevait en moyenne 2 transfusions sanguines par an et était hospitalisé en moyenne 3 fois par an. La moyenne annuelle des crises vaso-occlusives variait significativement avec l’âge du diagnostic (p = 0,02), les épisodes de fièvre (p = 0,005), le sexe (p = 0,007) et avec le niveau d’étude (p = 0,02) et la profession du responsable du ménage (p = 0,02). Les patients drépanocytaires diagnostiqués au plus tard à 1 année de vie présentaient en moyenne moins de crises vaso-occlusives par an que ceux qui étaient diagnostiqués au-delà d’une année. De même que les patients ayant présenté moins d’épisodes de fièvre par an (1-3 épisodes/an) présentaient en moyenne moins de crises vaso-occlusives par an que ceux qui ont présenté plus d’épisodes fébriles. Les garçons présentaient en moyenne plus des crises vaso-occlusives que les filles. Les enfants drépanocytaires vivant dans les familles dont le responsable a un niveau universitaire, et cadre ou employé présentent en moyenne plus des crises vaso-occlusives que ceux qui ne l’étaient pas. La moyenne annuelle des crises vaso-occlusives ne variait pas significativement avec la prophylaxie et le niveau de connaissance sur les facteurs favorisant les crises vaso-occlusives. Cependant, la prophylaxie adéquate à la pénicilline orale et à l’acide folique n’existait dans aucune famille. La moyenne annuelle des transfusions sanguines variait significativement avec les épisodes de fièvre (p = 0,008), les enfants drépanocytaires qui ont trois épisodes de fièvre par an ou plus ont une moyenne des transfusions plus élevée que ceux qui en ont moins. La moyenne annuelle des transfusions ne variait pas significativement avec l’âge du diagnostic, la prophylaxie, le sexe, et ni avec le niveau d’étude ou la profession du responsable du ménage.

**Tableau 3:** Description des caractéristiques médicales du premier enfant drépanocytaire de la famille (n= 50)

Caractéristiques médicales des patients drépanocytaires	Médiane (min – max)	N	%
**Age de diagnostic (année)**	1 (naiss – 15)		
A la naissance		18	36
Au cours des 6 premiers mois de vie		2	4
Entre 7 et 59 mois d’âge		27	54
Après 59 mois d’âge		3	6
**Base du diagnostic posé**			
Clinique uniquement		21	42
Test de falciformation		10	20
Electrophorèse de l’hémoglobine		19	38
**Nombre estimé d’épisodes des crises vaso-occlusives par an**	3 (0 – 10)		
Aucune		5	10
1 – 2		15	30
3 ou plus		30	60
**Nombre estimé d’épisodes de fièvre par an**	4 (0 – 7)		
Aucun		4	8
1 – 3		8	16
4 ou plus		38	76
**Nombre estimé d’épisodes des transfusions sanguines par an**	2 (0 – 6)		
Aucune		4	8
1 – 2		37	74
3 ou plus		9	18
**Nombre estimé d’épisodes d’hospitalisations par an**	3 (0 - 8)		
Aucune		2	4
1 – 2		22	44
3 ou plus		26	52

**Tableau 4:** Répartition de la moyenne du nombre des crises vaso-occlusives et des transfusions sanguines reçues par le premier enfant drépanocytaire en fonction des caractéristiques sociodémographiques des familles et les niveaux des connaissances des enquêtés

	**Moyenne des CVO/an**			**Moyenne des transfusions sanguine/an**	
	**n**	**Moy (DS)**	**p-value**	**Moy (DS)**	**p-value**
**Total de l’échantillon**		3,4 (2,58)		1,9 (1,52)	
**Age de diagnostic (année)**	50			**0,44**	**0,0249**
Naissance	18	4 (1,86)		2,28 (1,3)	
0 - 6 mois	2	2 (2,82)		1,5 (0,72)	
7 mois – 5 ans	27	3,03 (3,01)		1,7 (1,6)	
≥ 10 ans		3(2)		1 (1)	
**Observation de la prophylaxie contre** **l’anémie et les infections**	3			**0,66**	**0,159**
Acide folique et amoxicilline régulièrement ^+^	0	0		0	
Acide folique et amoxicilline irrégulièrement	1	8 (0, 0)		1 (0, 0)	
Acide folique seul, mais irrégulièrement^+^	13	4 (3,56)		2 (1,63)	
Antidouleurs	17	2,82 (1,50)		2 (1,13)	
Produits indigènes	2	1,50 (0,70)		0,5 (0,70)	
Aucune prophylaxie		2,17 (1,42)		2 (1,7)	
**Nombre estimé d’épisodes de fièvre par an**	17		**0,005**	**0,008**	
1 – 3	22	2,12 (1,40)		1,25 (1,66)	
4 ou plus		4,36 (2,80)		2,5 (0,68)	
**Sexe**	28		**0,007**		**0,44**
Masculin	25	4,32 (3,13)		2,04 (1,53)	
Féminin		2,40 (1,38)		1,72 (1,42)	
**Niveau de connaissance sur** **les facteurs favorisants les crises**	25		0,32		
Bon	4	2,5 (1,73)			
Assez bon	11	3,82 (2,22)			
Passable	14	2,42 (1,34)			
Nul	21	3,90 (3,33)			
**Niveau d’étude du responsable du ménage**			**0,022**		**0,60**
Primaire complète ou non	12	2,50 (2,84)		1,58 (1,78)	
Secondaire complète ou non	25	2,90 (2,21)		2,08 (1,38)	
Universitaire complète ou non	13	5,0 (2,45)		1,76 (1,36)	
**Profession du responsable de la famille**			**0,016**		**0,49**
Cadre	4	5,75 (2,06)		1,75 (1,50)	
Commerce	13	2,38 (2,14)		1,85 (1,46)	
Cultivateur	2	3 (0,0)		1 (0,0)	
Employé	12	5 (3,14)		2,5 (1,62)	
Sans emploi	19	2,57 (2,03)		1,63 (1,42)	

### Impact de la drépanocytose sur les familles enquêtées

La majorité de familles (60%) n’avait pas un revenu mensuel suffisant pour la prise en charge de leur (s) enfant (s) drépanocytaire (s). Septante pourcents des parents ne se sentaient coupable de la maladie de leur enfant. Parmi les répercussions sociales de la drépanocytose, le stress permanent est la répercussion la plus fréquemment rapporté avec 68% et suivi des divorces. La drépanocytose est vécue comme une situation normale pour 32% de familles, mais est vécue comme calvaire pour 22% et considérée comme mauvais sort pour 18% ou comme volonté divine dans 28% de familles. La majorité de familles souhaite la création d’un centre de référence pour la prise en charge de la drépanocytose à Mbujimayi et 94% acceptent de s’y abonner pour un montant annuel entre 50 – 100$.

## Discussion

Cette étude avait comme objectif d’évaluer le niveau des connaissances et pratiques des familles impliquées par la drépanocytose à Mbujimayi en RDC ainsi que l’impact de la drépanocytose dans leur quotidien. Les participants à l’enquête sont les responsables ou tuteurs répondants. Les résultats de cette enquête montrent l’existence de lacunes importantes au niveau des connaissances, des attitudes et des pratiques vis–à–vis de la drépanocytose ainsi qu’un impact négatif de cette maladie dans le quotidien des familles impliquées.

### Evaluation du niveau des connaissances des ménages impliqués par la drépanocytose

A la lumière d'autres recherches menées, notre étude a mis en évidence que 32% de tuteurs répondants ignoraient encore la cause de la maladie et ne connaissaient pas le mode de transmission de la drépanocytose et parmi eux certains croyaient à une transmission par mauvais sort. Les complications de la maladie n’ont été rapportées que par 6% (3/50) des enquêtés, 56% (28/50) ne connaissaient pas la durée de la maladie, 42% (21/50) n’avaient aucune connaissance des facteurs favorisants les crises vaso-occlusives. Ces résultats sont concordants avec ceux démontrés par Sangho H. [9] dans une étude transversale à passage unique au moyen d’un questionnaire administré à 360 mères d’au moins un enfant drépanocytaire. Dans cette étude, la cause de la drépanocytose semblait inconnue ou méconnue pour 58% (209/360) des individus interrogés. Par contre une étude transversale à passage unique réalisée au TOGO sur 210 individus dont 117 femmes et 93 hommes qui ont été interrogés, a démontré que 75% (157/210) connaissaient la cause de la maladie, 64% (134/210) connaissaient le pronostic, mais on note que 62% avaient une mauvaise connaissance sur les complications de la maladie [10]. L’étude menée au Togo a été réalisée à Lomé, la capitale du pays alors que notre étude a été menée dans une des provinces de la RDC. On sait que le niveau d’instruction est faible à l’intérieur du pays comparativement au niveau de la capitale. La majorité des répondants dans notre étude sont les mères des malades et elles ont un niveau d’instruction faible. Nos résultats peuvent s’expliquer par le lieu de l’étude et le type de répondants à l’enquête. La prise en charge de la maladie dans les familles interrogées n’est pas optimale. Ceci est néanmoins classique en Afrique subsaharienne. En effet, au Mali, sur base d’une enquête orale, le 1er recours en cas de maladie était la structure sanitaire (209/360; 58%) suivi de l’automédication (65/360; 18%) et des guérisseurs (50/360; 14%) [9]. De même Soumano et al. rapportent que les structures de soins représentaient le premier recours en cas de découverte de la maladie (194/360: 54%) et dans une moindre mesure, le recours aux guérisseurs (50/360; 14%) [9]. Le manque de connaissance de la maladie mais également des problèmes économiques et/ou d’accessibilité aux soins peuvent expliquer ce recours aux traitements traditionnels. Diarra Yé et al, au Burkina Fasso ont montré dans une série de 76 enfants drépanocytaires de 6 mois à 16 ans qui étaient hospitalisés, qu’aucun enfant ne recevait régulièrement la pénicilline en prophylaxie. Par contre une prophylaxie à l’acide folique associé souvent au fer était réalisée. Par ailleurs, une chimio prophylaxie antipaludique était assurée [11].

### La situation médicale des enfants drépanocytaires et l’impact de la maladie sur les familles impliquées

Il ressort de notre étude que la situation médicale des enfants drépanocytaires est préoccupante. Le diagnostic est posé au-delà de l’âge de 6 mois pour plus de la moitié des malades et sans diagnostic définitif mais bien clinique pour 42% des cas. La morbidité liée à la drépanocytose est sévère puisque annuellement chaque malade fait en moyenne 3 crises vaso-occlusives, 4 épisodes de fièvre, reçoit 2 transfusions sanguines et est hospitalisé à 3 reprises. L’absence d’un programme structuré de lutte contre la drépanocytose à Mbujimayi est probablement une des causes principales de cette situation. Il n’existe pas de programme qui inclut l’information/formation du personnel et de la communauté, et des moyens alloués à la lutte contre la drépanocytose à l’instar d’autres programmes notamment le VIH/SIDA. A partir de 2009, on observe une décroissance des décès de patients VIH jusqu’en 2012 parallèlement à l’introduction d’un programme sanitaire structuré [12]. Dans notre milieu, le diagnostic de la drépanocytose est rarement posé avant l’âge de 2 ans en dehors d’un dépistage néonatal systématique proposé à la population [13]. Dans l’étude réalisée au Mali, la moyenne des transfusions sanguines (1,9) était identique à celle de notre étude [14]. La fréquence d’épisodes infectieux a été rapportée à des valeurs de 45% (110/247) à Kinshasa [15]. Au Congo-Brazzaville, une étude a démontré une relation entre le nombre des crises vaso-occlusives et les crises palustres (49%), les infections bactériennes (25%) et ceci plus fréquemment chez les garçons [16].

Paradoxalement, on s’attendrait à ce que les enfants dont le responsable de famille a un niveau d’étude universitaire ou une fonction de cadre puissent faire moins de crises, mais les résultats observés sont à l’opposé. Il est rapporté une fréquence élevée des crises quand la consultation est tardive et que cette situation serait expliquée par le faible niveau d’instruction (69,7%) et de revenu chez la plupart des familles enquêtées (51,5%) [17]. Dans plusieurs études, le niveau d’études était associé à un bon contrôle de la maladie. Les patients drépanocytaires qui détiennent un diplôme universitaire ont signalé des effets positifs sur les domaines suivants de suivi médical: l’état physique, la vitalité, le bien-être, la fonction sociale et émotionnelle, la réduction des crises douloureuses et de l’état de santé en général [18]. Dans notre série, cette situation pourrait être expliquée par un revenu faible de la majorité de familles.

Quant à l’impact de la drépanocytose sur les familles enquêtées à Mbujimayi, nous notons dans notre série que la majorité présentait des difficultés financières. Ceci est lié à la situation socio-économique de la RDC en général et en particulier pour la ville de Mbujimayi. L’existence d’une seule entreprise minière pour toute la province, en faillite depuis plus de 5 ans, la pauvreté de la population et le chômage sont autant de facteurs qui entretiennent ces difficultés financières. Au niveau psychosocial, le stress permanent était l’impact majeur de la drépanocytose dans les familles. Certaines familles éprouvaient une instabilité allant jusqu’au divorce, aggravant ainsi d’avantage la situation de la prise en charge du malade. Bien que la situation semble catastrophique, la majorité de familles a émis le vœu d’une création d’un centre spécialisé de prise en charge de la drépanocytose à Mbujimayi et a manifesté la volonté d’y souscrire un abonnement annuel allant de 50 à 100$. Des études similaires appuient nos résultats. En RDC, les 2/3 de patients drépanocytaires ont un niveau de vie bas avec un revenu moyen de moins de 1 dollar américain par jour [14]. Cette proposition des parents parait raisonnable à l’instar d’une étude réalisée au Burkina Faso qui montre que le coût moyen d’une prise en charge en ambulatoire est estimé à environ 71$ par an et le coût moyen de la prise en charge en hospitalisation à environ 185$. La drépanocytose constitue donc un poids économique considérable pour nos patients et surtout les familles. Une prise en charge adéquate et structurée mérite d’être envisagée. L’implication des pouvoirs politiques pour la mise en place de protocoles bien codifiés et d’une subvention pour la prise en charge de cette affection est indispensable [19].

## Conclusion

Cette étude met en évidences les lacunes au niveau des connaissances et pratiques des familles impliquées dans la drépanocytose à Mbujimayi en RDC. La situation de cette maladie reste préoccupante dans cette contrée de la RDC que ce soit au niveau de la morbidité ou de son impact sur les familles impliquées. Des mesures de lutte impliquant des actions de sensibilisation et des actions médicales sont nécessaires pour réduire la morbi-mortalité de cette maladie. Nous proposons de mettre en place un centre de référence pour une prise en charge structurée des patients qui inclut également un dépistage néonatal et une systématisation de l’antibioprophylaxie et, de la vaccination; ce centre offrira une possibilité de financement forfaitaire annuel pour cette prise en charge.

### Etat des connaissances actuelles sur le sujet

- La drépanocytose est une des maladies héréditaires récessives les plus fréquentes à travers le monde et en particulier en Afrique sub-saharienne; on estime que 7% de la population mondiale sont porteurs d’une anomalie de l’hémoglobine (anémie falciforme et la thalassémie) et chaque année, 300 000 enfants naissent avec la maladie dont les 2/3 en Afrique sub-saharienne; il est rapporté que près de plus de 50% d’enfants atteints par la drépanocytose meurent avant leur cinquième anniversaire s’ils ne sont pas suivis médicalement.
- Le niveau de connaissance, l’attitude et l’impact de la drépanocytose dans les familles impliquées varient d’une famille à une autre, et d’un milieu culturel à un autre; ils renvoient aux environnements sociaux, psychosociaux, financiers ou matériels;
- Des études réalisées dans d’autres villes de la RDC sur la drépanocytose montrent une grande prévalence de cette maladie parmi les populations originaires du Kasaï, et aucune étude n’est réalisée localement au Kasaï.

### Contribution de notre étude à la connaissance

- La première littérature sur la drépanocytose à Mbujimayi au Kasaï oriental en RDC;
- Notre étude met en lumière la gravité de la situation de la drépanocytose dans les familles impliquées à Mbujimayi et l’importance de sensibiliser davantage les individus et les communautés sur cette maladie, à renforcer la prévention primaire, à réduire l’incidence de la drépanocytose, ainsi que la morbidité et la mortalité relatives à cette maladie, et à améliorer la qualité de vie des malades et des familles;
- De façon globale, notre étude suggère des interventions préventives précoces, qui visent non seulement à une transmission des connaissances et des pratiques vis-à-vis de la drépanocytose afin de réduire son impact au niveau des familles impliquées, mais surtout de mettre en place un programme actif de lutte contre la drépanocytose avec des actions synergiques à deux volets, l’éducation et intervention médicale, qui peuvent garantir la réussite du dit programme.

## Conflits d’intérêts

Les auteurs ne déclarent aucun conflit d'intérêts.

## Remerciements

Nous remercions chaleureusement toute l'Equipe de la Faculté de médecine et les étudiants de deuxième master de médecine de l’université de Mbujimayi pour leur contribution multiforme (amendement du protocole, formation des enquêteurs, collecte et encodage des données, vérification des questionnaires, etc...). Nos remerciements s’adressent également à tout le personnel de la Clinique Pédiatrique de Mbujimayi d’avoir servi de référence pour tous les patients drépanocytaires rencontrés sur terrain.
